# Benzyl-1,2,4-triazoles as CB_**1**_ Cannabinoid Receptor Ligands: Preparation and* In Vitro* Pharmacological Evaluation

**DOI:** 10.1155/2016/1257098

**Published:** 2016-03-31

**Authors:** Laura Hernandez-Folgado, Juan Decara, Fernando Rodríguez de Fonseca, Pilar Goya, Nadine Jagerovic

**Affiliations:** ^1^Instituto de Química Médica, CSIC, Juan de la Cierva 3, 28006 Madrid, Spain; ^2^Centros de Investigación en Red (CIBER) Fisiopatología de la Obesidad y Nutrición, Instituto de Salud Carlos III, CB06/03, 28029 Madrid, Spain; ^3^Unidad Gestión Clínica de Salud Mental, Instituto de Investigación Biomédica de Málaga (IBIMA), Hospitales Universitarios Regional y Virgen de la Victoria de Málaga, Universidad de Málaga, 29071 Málaga, Spain

## Abstract

In a previous study, we have identified 3-alkyl-1,5-diaryl-1*H*-1,2,4-triazoles to be a novel class of cannabinoid type 1 receptor (CB_1_R) antagonists. In order to expand the number of cannabinoid ligands with a central 1,2,4-triazole scaffold, we have synthesized a novel series of 1-benzyl-1*H*-1,2,4-triazoles, and some of them were evaluated by CB_1_R radioligand binding assays. Compound** 12a** showed the most interesting pharmacological properties, possessing a CB_1_R affinity in the nanomolar range.

## 1. Introduction

Due to the potential therapeutic effects of cannabinoids that include antiemetic, analgesic, antiglaucoma, obesity treatment, alcoholism, bronchodilatation, and inflammation, a considerable number of cannabinoid ligands have been reported in recent years [1]. Their effects are mediated through G-protein coupled cannabinoid receptors, which are part of the endocannabinoid system (ECS) [2]. So far, two types of cannabinoid receptors, designated as CB_1_R and CB_2_R, have been well characterized, and three putative cannabinoid receptors, GPR55, GPR18, and GPR119, have been also proposed [3]. CB_1_R has been found in the peripheral and central nervous system, and CB_2_R is mainly present in the immune system. Cannabinoid ligands belong to families of diverse structural classes such as eicosanoids, classical and nonclassical ligands related to Δ^9^-tetrahydrocannabinol (THC), and heterocycles. Among the heterocycles family, pyrazoles [4] and aminoalkylindoles [5] are the most representative ligands.

**Figure 1:**
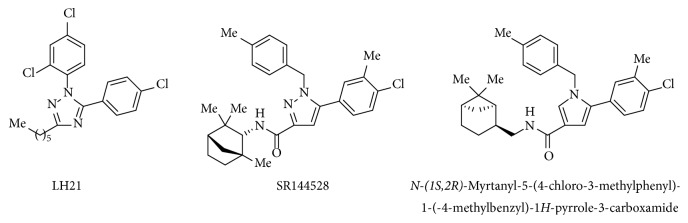
Structure of the CB_1_R antagonist LH21 and the CB_2_R antagonists SR144528 and* N*-(*1S*,*2R*)-myrtanyl-5-(4-chloro-3-methylphenyl)-1-(-4-methylbenzyl)-1*H*-pyrrole-3-carboxamide.

In our early research program, it was found that triazole motif was an attractive scaffold for cannabinoid activity [6]. We reported that the CB_1_R antagonist 5-(4-chlorophenyl)-1-(2,4-dichlorophenyl)-3-hexyl-1*H*-1,2,4-triazole (LH21) exhibited antiobesity activity in* in vivo* assays (Figure 1) [7–9]. Pyrazole [10] and pyrrole [11] cannabinoid ligands bearing a benzyl substituent on position N1 have been reported in the literature as CB_2_R antagonists (Figure 1). This prompted us to extend our previous investigation by synthesizing a series of 3-alkyl-5-aryl-1-benzyl-1*H*-1,2,4-triazoles in order to establish structure-activity relationships.

We describe herein the synthesis of new benzyl-1,2,4-triazoles [12] and present initial results from radioligand binding assays as part of our investigation on cannabinoid active compounds.

## 2. Materials and Methods

### 2.1. Chemistry

#### 2.1.1. General

**Scheme 1:**
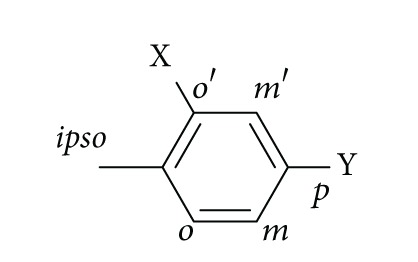


All reagents and solvents were used as commercially received. EtOH was dried over magnesium. TLC was carried out by precoated silica-gel 60 F254 plates (Merck) and detection by UV light (254 nm). Flash-column chromatography was carried out by Kieselgel 60 (230–400 mesh; Merck). Medium pressure chromatography (MPLC) was carried out by Flash Master Personal system with prepacked silica-gel cartridges. The purity of the final compounds was determined by elemental analysis or analytical HPLC. Elemental analysis was performed on a Heraeus CHN-O rapid analyzer. Analyses indicated by the symbols of the elements or functions were within ±0.4% of the theoretical values, except compound** 6**. Analytical HPLC was run on a Waters 6000 with Delta Pak C 18.5 mm, 300 Ǻ (3.9 × 150 mm) column, using an eluent Acetonitrile/H_2_O (0.05% H_3_PO_4_ + 0.04% TEA) in the proportion indicated in each case; flow rate used was 1 mL/min and the UV absorption was detected at a wavelength of 254 nm. HPLC analyses were within ≥90% of purity, except compound** 11b** (81% purity). The mass spectra (electrospray positive mode) were determined on a MSD-Series 1100 Hewlett Packard instrument. Melting points (uncorrected) were determined with a Reichert Jung Thermovar apparatus. ^1^H and ^13^C NMR spectra were recorded on a Gemini 200, Varian 300 and 400 unity spectrometers using TMS as the internal standard. All chemical shifts are reported in ppm. For the assignment of the protons and carbons of the aromatic rings Scheme 1 is used.

#### 2.1.2. General Procedure for the Synthesis of** 1** and** 2**

To a suspension of the corresponding nitrile (10 equiv) in dry EtOH (30–75 mL) NaOMe (1 equiv) was added. It was stirred at room temperature under N_2_ atmosphere for 48 h. Afterwards, ammonium chloride (10 equiv) was added, and the stirring was maintained for 24 more hours. Then, unreacted ammonium chloride was filtered off and the solvent was evaporated from the liquid layer. The white solid obtained was washed with Et_2_O, dried, and used in the next step without further purification.

*4-Chlorobenzimidamide Hydrochloride (* ***1***). Compound** 1** was prepared from 4-chlorobenzonitrile (10.00 g, 72.7 mmol), NaOMe (393 mg, 7.3 mmol), and ammonium chloride (3.90 g, 72.7 mmol). Yield: 4.34 g of** 1** (31%) as a white solid. Mp = 246-247°C (236–240°C (EtOH)). [13] ^1^H-NMR (CD_3_OD) *δ*: 8.02 (d, 2H, *J* = 9.0 Hz, H*o*); 7.84 (d, 2H, *J* = 9.0 Hz, H*m*). ^13^C-NMR (CD_3_OD) *δ*: 167.6 C=NH; 141.4 C*p*; 130.8 and 130.7 C*o* and C*m*; 128.2 C*ipso*. MS (ES^+^)* m/z*: 155 (100%) [M+H]^+^.

*4-Amidinopyridinium Hydrochloride (* ***2***). Compound** 2** was prepared from 4-cyanopyridine (2.50 g, 24.0 mmol), NaOMe (130 mg, 2.4 mmol), and ammonium chloride (1.28 g, 24.0 mmol). Yield: 3.30 g of** 2** (87%) as a white solid. Mp = 248-249°C. ^1^H-NMR (CD_3_OD) *δ*: 8.93 (d, 2H, *J* = 6.2 Hz, H*m*); 7.87 (d, 2H, *J* = 6.2 Hz, H*o*). ^13^C-NMR (CD_3_OD) *δ*: 166.9 C=NH; 151.7 C*m*; 138.1 C*ipso*; 123.2 C*o*. MS (ES^+^)* m/z*: 122 (100%) [M+H]^+^.

#### 2.1.3. General Procedure for the Synthesis of** 3**–**5**

To a solution of the corresponding amidinium salt (1.5 equiv) in dry EtOH (10–45 mL), NaOMe (1 equiv) in 10 mL of dry EtOH was added. The suspension was stirred at room temperature for 1 h. Then, the solid formed was filtered on Celite. Octanoic hydrazide (2 equiv) was added to the liquid layer and the mixture was stirred under reflux for 46–49 h. After cooling the reaction mixture, solvent was removed* in vacuo*. The residue was dissolved in CH_2_Cl_2_ and washed with water (3 × 20 mL). The organic layer was dried over anhydrous Na_2_SO_4_ and the solvent was removed* in vacuo*. The obtained residue was purified by MPLC using cyclohexane/EtOAc (3 : 1) as eluent, except for compound** 5** where cyclohexane/EtOAc (3 : 1 to 4 : 1) was used. 

*3-Heptyl-5-phenyl-1H-1,2,4-triazole (* ***3***). Compound** 3** was prepared from benzamidine hydrochloride hydrate (857 mg, 5.5 mmol), octanoic hydrazide (581 mg, 3.6 mmol), and NaOMe (394 mg, 7.3 mmol). Yield: 683 mg of** 3** (78%) as a transparent oil. Mp = 129–132°C oxalate (to a solution of the free base in Et_2_O, a solution of oxalic acid in EtOAc was added; the white solid was filtered off, washed with EtOAc, and dried). ^1^H-NMR (CDCl_3_) *δ*: 10.65 (bs, 1H, NH); 7.96 (m, 2H, H*o*); 7.34 (m, 3H, H*m* and H*p*); 2.69 (t, 2H, *J* = 7.7 Hz, C*H*_2_CH_2_CH_2_CH_2_CH_2_CH_2_CH_3_); 1.65 (p, 2H, *J* = 7.7 Hz, CH_2_C*H*_2_CH_2_CH_2_CH_2_CH_2_CH_3_); 1.16 (m, 8H, CH_2_CH_2_C*H*_2_C*H*_2_C*H*_2_C*H*_2_CH_3_); 0.79 (bt, 3H, *J* = 6.3 Hz, CH_3_). ^13^C-NMR (CDCl_3_) *δ*: 160.4 and 160.0 C3 and C5; 129.8 C*ipso*; 129.5 C*p*; 128.6 C*m*; 126.4 C*o*; 31.5 CH_2_CH_2_CH_2_CH_2_ *C*H_2_CH_2_CH_3_; 29.1 CH_2_CH_2_ *C*H_2_CH_2_CH_2_CH_2_CH_3_; 28.8 CH_2_CH_2_CH_2_ *C*H_2_CH_2_CH_2_CH_3_; 28.1 CH_2_ *C*H_2_CH_2_CH_2_CH_2_CH_2_CH_3_; 27.0* C*H_2_CH_2_CH_2_CH_2_CH_2_CH_2_CH_3_; 22.5 CH_2_CH_2_CH_2_CH_2_CH_2_ *C*H_2_CH_3_; 13.9 CH_3_. MS (ES^+^)* m/z*: 244 (100%) [M+H]^+^. Anal (C_15_H_21_N_3_·C_2_H_2_O_4_) % calculated (% found) C: 61.25 (61.41); H: 6.95 (7.12); N: 12.60 (12.62).

*5-(4-Chlorophenyl)-3-heptyl-1H-1,2,4-triazole (* ***4***). Compound** 4** was prepared from** 1** (1.00 g, 5.2 mmol), octanoic hydrazide (549 mg, 3.5 mmol), and NaOMe (375 mg, 7.0 mmol). Yield 392 mg of** 4** (40%) as a white solid. Mp = 108–111°C. ^1^H-NMR (CDCl_3_) *δ*: 7.91 (d, 2H, *J* = 8.6 Hz, H*o*); 7.34 (d, 2H, *J* = 8.6 Hz, H*m*); 2.72 (t, 2H, *J* = 7.6 Hz, C*H*_2_CH_2_CH_2_CH_2_CH_2_CH_2_CH_3_); 1.68 (p, 2H, *J* = 7.6 Hz, CH_2_C*H*_2_CH_2_CH_2_CH_2_CH_2_CH_3_); 1.20 (m, 8H, CH_2_CH_2_C*H*_2_C*H*_2_C*H*_2_C*H*_2_CH_3_); 0.82 (bt, 3H, *J* = 6.5 Hz, CH_3_). ^13^C-NMR (CDCl_3_) *δ*: 160.4 and 159.5 C3 and C5; 135.5 C*ipso*; 128.9 C*m*; 128.7 C*p*; 127.7 C*o*; 31.6 CH_2_CH_2_CH_2_CH_2_ *C*H_2_CH_2_CH_3_; 29.2 CH_2_CH_2_ *C*H_2_CH_2_CH_2_CH_2_CH_3_; 28.8 CH_2_CH_2_CH_2_ *C*H_2_CH_2_CH_2_CH_3_; 28.0 CH_2_ *C*H_2_CH_2_CH_2_CH_2_CH_2_CH_3_; 26.9* C*H_2_CH_2_CH_2_CH_2_CH_2_CH_2_CH_3_; 22.5 CH_2_CH_2_CH_2_CH_2_CH_2_ *C*H_2_CH_3_; 14.0 CH_3_. MS (ES^+^)* m/z*: 278 (100%) [M+H]^+^. Anal (C_15_H_20_ClN_3_) % calculated (% found) C: 64.85 (65.09); H: 7.26 (7.42); N: 15.13 (15.35).

*4-(3-Heptyl-1H-1,2,4-triazol-5-yl)pyridine (* ***5*** *) and N*′*-[imino(pyridin-4-yl)methyl]octa-nehydrazide (* ***6***). Compound** 5** was prepared from** 2** (2.00 g, 12.7 mmol), octanoic hydrazide (1.35 g, 8.5 mmol), and NaOMe (918 mg, 17.0 mmol). Yield: 459 mg of** 5** (23%) as a white solid and 1.61 g of** 6** (45%) as a white solid.** 5**: Mp = 109–112°C. ^1^H-NMR (CDCl_3_) *δ*: 8.69 (d, 2H, *J* = 6.1 Hz, H*m*); 8.06 (d, 2H, *J* = 6.1 Hz, H*o*); 2.85 (t, 2H, *J* = 7.7 Hz, C*H*_2_CH_2_CH_2_CH_2_CH_2_CH_2_CH_3_); 1.78 (p, 2H, *J* = 7.7 Hz, CH_2_C*H*_2_CH_2_CH_2_CH_2_CH_2_CH_3_); 1.21 (m, 8H, CH_2_CH_2_C*H*_2_C*H*_2_C*H*_2_C*H*_2_CH_3_); 0.81 (bt, 3H, *J* = 6.7 Hz, CH_3_). ^13^C-NMR (CDCl_3_) *δ*: 159.4 and 159.2 C3 and C5; 149.4 C*m*; 139.5 C*ipso*; 121.0 C*o*; 31.5 CH_2_CH_2_CH_2_CH_2_ *C*H_2_CH_2_CH_3_; 29.1 CH_2_CH_2_ *C*H_2_CH_2_CH_2_CH_2_CH_3_; 28.8 CH_2_CH_2_CH_2_ *C*H_2_CH_2_CH_2_CH_3_; 28.1 CH_2_ *C*H_2_CH_2_CH_2_CH_2_CH_2_CH_3_; 26.7* C*H_2_CH_2_CH_2_CH_2_CH_2_CH_2_CH_3_; 22.5 CH_2_CH_2_CH_2_CH_2_CH_2_ *C*H_2_CH_3_; 13.9 CH_3_. MS (ES^+^)* m/z*: 245 (100%) [M+H]^+^. Anal (C_14_H_20_N_4_) % calculated (% found) C: 68.82 (68.71); H: 8.25 (8.36); N: 22.93 (22.78).** 6**: Mp = 135–138°C. ^1^H-NMR (CD_3_OD) *δ*: 8.67 (d, 2H, *J* = 6.1 Hz, H*m*); 7.94 (d, 2H, *J* = 6.1 Hz, H*o*); 2.41 (t, 2H, *J* = 7.4 Hz, C*H*_2_CH_2_CH_2_CH_2_CH_2_CH_2_CH_3_); 1.76 (m, 2H, CH_2_C*H*_2_CH_2_CH_2_CH_2_CH_2_CH_3_); 1.41 (m, 8H, CH_2_CH_2_C*H*_2_C*H*_2_C*H*_2_C*H*_2_CH_3_); 0,99 (bt, 3H, *J* = 6.0 Hz, CH_3_). ^13^C-NMR (CD_3_OD) *δ*: 172.7 CONH; 151.7 C=NH; 150.4 C*m*; 144.1 C*ipso*; 122.9 C*o*; 35.7* C*H_2_CH_2_CH_2_CH_2_CH_2_CH_2_CH_3_; 32.9 CH_2_CH_2_CH_2_CH_2_ *C*H_2_CH_2_CH_3_; 30.4 and 30.2 CH_2_CH_2_ *C*H_2_ *C*H_2_CH_2_CH_2_CH_3_; 27.1 CH_2_ *C*H_2_CH_2_CH_2_CH_2_CH_2_CH_3_; 23.7 CH_2_CH_2_CH_2_CH_2_CH_2_ *C*H_2_CH_3_; 14.4 CH_3_. MS (ES^+^)* m/z*: 263 (100%) [M+H]^+^. Anal (C_14_H_22_N_4_O·1/2HCl) % calculated (% found) C: 59.93 (59.10); H: 8.08 (8.11); N: 19.97 (20.45).

Intermediate** 6** (1.00 g, 3.6 mmol) in dry EtOH (20 mL) reacted by refluxing with NaOMe (1.03 g, 19.0 mmol) for 4 days. Under this procedure,** 5** was obtained in 78% yield (676 mg).

#### 2.1.4. General Procedure for the Synthesis of** 7a**–**15a** and** 7b**–**15b**

To a solution of the 3,5-disubstituted triazole (1 equiv) in 40% NaOH aq solution (3–5 mL) and toluene (7–10 mL) (Bu)_4_NBr (0.05 equiv) were first added, and later the corresponding alkylating agent (1 equiv) was added. The reaction mixture was stirred at 80–90°C (bath temperature) for the reaction time indicated. Afterwards, organic layer was separated and the aqueous layer was extracted with CH_2_Cl_2_ (3 × 25 mL). The combined organic layers were dried over anhydrous Na_2_SO_4_ and the solvent was removed* in vacuo*. The residue was purified by MPLC [cyclohexane/EtOAc (9 : 1)], except for compound** 13a**, which was purified by flash chromatography [CH_2_Cl_2_ → CH_2_Cl_2_/MeOH (60 : 1)].

*1-Benzyl-5-heptyl-3-phenyl-1H-1,2,4-triazole (* ***7a*** *) and 1-Benzyl-3-heptyl-5-phenyl-1H-1,2,4-triazole (* ***7b***). Compounds** 7a** and** 7b** were prepared from** 3** (100 mg, 0.4 mmol), benzyl bromide (52 *μ*L, 0.4 mmol), and (Bu)_4_NBr (6 mg, 0.02 mmol); reaction time: 1.5 h. Yield: 106 mg of** 7a** (78%) as a transparent oil, and 13 mg of** 7b** (9%) as a transparent oil.** 7a**: ^1^H-NMR (CDCl_3_) *δ*: 8.08 (m, 2H, Ph); 7.40 (m, 3H, Ph); 7.31 (m, 3H, Bn); 7.18 (m, 2H, Bn); 5.34 (s, 2H, CH_2_Ph); 2.69 (t, 2H, *J* = 7.8 Hz, C*H*_2_CH_2_CH_2_CH_2_CH_2_CH_2_CH_3_); 1.64 (p, 2H, *J* = 7.5 Hz, CH_2_C*H*_2_CH_2_CH_2_CH_2_CH_2_CH_3_); 1.22 (m, 8H, CH_2_CH_2_C*H*_2_C*H*_2_C*H*_2_C*H*_2_CH_3_); 0.85 (bt, 3H, *J* = 6.1 Hz, CH_3_). ^13^C-NMR (CDCl_3_) *δ*: 160.8 C3; 157.0 C5; 135.7 C*ipso* Bn; 131.2 C*ipso* Ph; 128.9 C*m* Bn and C*p* Ph; 128.5 C*m* Ph; 126.9 C*o* Bn; 126.3 C*o* Ph; 128.1 C*p* Bn; 52.1 (CH_2_Ph); 31.6 CH_2_CH_2_CH_2_CH_2_ *C*H_2_CH_2_CH_3_; 29.2 CH_2_CH_2_ *C*H_2_CH_2_CH_2_CH_2_CH_3_; 28.8 CH_2_CH_2_CH_2_ *C*H_2_CH_2_CH_2_CH_3_; 27.8 CH_2_ *C*H_2_CH_2_CH_2_CH_2_CH_2_CH_3_; 26.2* C*H_2_CH_2_CH_2_CH_2_CH_2_CH_2_CH_3_; 22.5 CH_2_CH_2_CH_2_CH_2_CH_2_ *C*H_2_CH_3_; 14.0 CH_3_. MS (ES^+^)* m/z*: 334 (100%) [M+H]^+^. Anal (C_22_H_27_N_3_) % calculated (% found) C: 79.24 (79.35); H: 8.16 (8.40); N: 12.60 (12.64).** 7b**: ^1^H-NMR (CDCl_3_) *δ*: 7.53 (m, 2H, Ph); 7.41 (m, 3H, Ph); 7.30 (m, 3H, Bn); 7.14 (m, 2H, Bn); 5.35 (s, 2H, CH_2_Ph); 2.75 (t, 2H, *J* = 7.7 Hz, C*H*_2_CH_2_CH_2_CH_2_CH_2_CH_2_CH_3_); 1.78 (p, 2H, *J* = 7.7 Hz, CH_2_C*H*_2_CH_2_CH_2_CH_2_CH_2_CH_3_); 1.23 (m, 8H, CH_2_CH_2_C*H*_2_C*H*_2_C*H*_2_C*H*_2_CH_3_); 0.85 (bt, 3H, *J* = 6.4 Hz, CH_3_). ^13^C-NMR (CDCl_3_) *δ*: 164.1 C3; 155.3 C5; 136.1 C*ipso* Bn; 130.2 C*ipso* Ph; 128.9 C*m* Bn; 128.8 and 128.7 C*o* and C*m* Ph; 126.7 C*o* Bn; 127.9 C*p* Bn and C*p* Ph; 52.4 CH_2_Ph; 31.8 CH_2_CH_2_CH_2_CH_2_ *C*H_2_CH_2_CH_3_; 29.3 CH_2_CH_2_ *C*H_2_CH_2_CH_2_CH_2_CH_3_; 29.0 CH_2_CH_2_CH_2_ *C*H_2_CH_2_CH_2_CH_3_; 28.5 CH_2_ *C*H_2_CH_2_CH_2_CH_2_CH_2_CH_3_; 28.3* C*H_2_CH_2_CH_2_CH_2_CH_2_CH_2_CH_3_; 22.6 CH_2_CH_2_CH_2_CH_2_CH_2_ *C*H_2_CH_3_; 14.1 CH_3_. MS (ES^+^)* m/z*: 334 (100%) [M+H]^+^. Anal (C_22_H_27_N_3_) % calculated (% found) C: 79.24 (79.10); H: 8.16 (7.95); N: 12.60 (12.42).

*1-(4-Chlorobenzyl)-5-heptyl-3-phenyl-1H-1,2,4-triazole (* ***8a*** *) and 1-(4-Chlorobenzyl)-3-heptyl-5-phenyl-1H-1,2,4-triazole (* ***8b***). Compounds** 8a** and** 8b** were prepared from** 3** (73 mg, 0.3 mmol), 4-chlorobenzyl chloride (48 mg, 0.3 mmol), and (Bu)_4_NBr (6 mg, 0.02 mmol); reaction time: 11 h. Yield: 96 mg of** 8a** (87%) as a yellow solid and 10 mg of** 8b** (9%) as a transparent oil.** 8a**: ^1^H-NMR (CDCl_3_) *δ*: 8.07 (m, 2H, H*o* Ph); 7.37 (m, 3H, H*m* and H*p* Ph); 7.29 (d, 2H, *J* = 8.4 Hz, H*m* Bn); 7.11 (d, 2H, *J* = 8.4 Hz, H*o* Bn); 5.27 (s, 2H, CH_2_Ar); 2.67 (t, 2H, *J* = 7.7 Hz, C*H*_2_CH_2_CH_2_CH_2_CH_2_CH_2_CH_3_); 1.69 (p, 2H, *J* = 7.7 Hz, CH_2_C*H*_2_CH_2_CH_2_CH_2_CH_2_CH_3_); 1.24 (m, 8H, CH_2_CH_2_C*H*_2_C*H*_2_C*H*_2_C*H*_2_CH_3_); 0.85 (ta, 3H, *J* = 6.9 Hz, CH_3_). ^13^C-NMR (CDCl_3_) *δ*: 161.0 C3; 156.9 C5; 134.2 C*ipso* Bn; 134.0 C*p* Bn; 131.1 C*ipso* Ph; 129.0 and 128.3 C*o* Bn, C*m* Bn and C*p* Ph; 128.5 C*m* Ph; 126.2 C*o* Ph; 51.3 CH_2_Ar; 31.5 CH_2_CH_2_CH_2_CH_2_ *C*H_2_CH_2_CH_3_; 29.2 CH_2_CH_2_ *C*H_2_CH_2_CH_2_CH_2_CH_3_; 28.8 CH_2_CH_2_CH_2_ *C*H_2_CH_2_CH_2_CH_3_; 27.7 CH_2_ *C*H_2_CH_2_CH_2_CH_2_CH_2_CH_3_; 26.1* C*H_2_CH_2_CH_2_CH_2_CH_2_CH_2_CH_3_; 22.5 CH_2_CH_2_CH_2_CH_2_CH_2_ *C*H_2_CH_3_; 14.0 CH_3_. MS (ES^+^)* m/z*: 368 (100%) [M+H]^+^. Anal (C_22_H_26_ClN_3_) % calculated (% found) C: 71.82 (71.75); H: 7.12 (6.98); N: 11.42 (11.63).** 8b**: ^1^H-NMR (CDCl_3_) *δ*: 7.50 (m, 2H, H Ph); 7.43 (m, 3H, Ph); 7.29 (d, 2H, *J* = 8.4 Hz, H*m* Bn); 7.06 (d, 2H, *J* = 8.4 Hz, H*o* Bn); 5.30 (s, 2H, CH_2_Ar); 2.75 (t, 2H, *J* = 7.5 Hz, C*H*_2_CH_2_CH_2_CH_2_CH_2_CH_2_CH_3_); 1.77 (p, 2H, *J* = 7.5 Hz, CH_2_C*H*_2_CH_2_CH_2_CH_2_CH_2_CH_3_); 1.23 (m, 8H, CH_2_CH_2_C*H*_2_C*H*_2_C*H*_2_C*H*_2_CH_3_); 0.85 (ta, 3H, *J* = 6.2 Hz, CH_3_). ^13^C-NMR (CDCl_3_) *δ*: 164.6 C3; 155.5 C5; 134.7 C*ipso* Bn; 133.8 C*p* Bn; 130.1 C*p* Ph; 129.0 C*m* Bn; 128.8 C*o* Bn; 128.6 C*m* Ph; 128.1 C*o* Ph; 128.0 C*ipso* Ph; 51.7 CH_2_Ar; 31.8 CH_2_CH_2_CH_2_CH_2_ *C*H_2_CH_2_CH_3_; 29.4 CH_2_CH_2_ *C*H_2_CH_2_CH_2_CH_2_CH_3_; 29.0 CH_2_CH_2_CH_2_ *C*H_2_CH_2_CH_2_CH_3_; 28.5* C*H_2_ *C*H_2_CH_2_CH_2_CH_2_CH_2_CH_3_; 22.6 CH_2_CH_2_CH_2_CH_2_CH_2_ *C*H_2_CH_3_; 14.1 CH_3_. MS (ES^+^)* m/z*: 368 (100%) [M+H]^+^. HPLC: Acetonitrile/H_2_O 95 : 5, *t*_*R*_ = 28.5 min (99% purity).

*1-(2,4-Dichlorobenzyl)-5-heptyl-3-phenyl-1H-1,2,4-triazole (* ***9a*** *) and 1-(2,4-Dichloro-benzyl)-3-heptyl-5-phenyl-1H-1,2,4-triazole (* ***9b***). Compounds** 9a** and** 9b** were prepared from** 3** (100 mg, 0.4 mmol), 2,4-dichlorobenzyl chloride (57 *μ*L, 0.4 mmol), and (Bu)_4_NBr (6 mg, 0.02 mmol); reaction time: 5 h. Yield: 123 mg of** 9a** (74%) as a white solid and 12 mg of** 9b** (7%) as a transparent oil.** 9a**: Mp = 70–73°C. ^1^H-NMR (CDCl_3_) *δ*: 8.11 (m, 2H, H*o* Ph); 7.42 (m, 4H, H*m*′ Bn, H*m* and H*p* Ph); 7.19 (dd, 1H,* J* = 8.3 Hz and 1.6 Hz, H*m* Ph); 6.85 (d, 1H, *J* = 8.3 Hz, H*o* Bn); 5.41 (s, 2H, CH_2_Ar); 2.73 (t, 2H, *J* = 7.7 Hz, C*H*_2_CH_2_CH_2_CH_2_CH_2_CH_2_CH_3_); 1.73 (p, 2H, *J* = 7.7 Hz, CH_2_C*H*_2_CH_2_CH_2_CH_2_CH_2_CH_3_); 1.27 (m, 8H, CH_2_CH_2_C*H*_2_C*H*_2_C*H*_2_C*H*_2_CH_3_); 0.88 (bt, 3H, *J* = 6.4 Hz, CH_3_). ^13^C-NMR (CDCl_3_) *δ*: 161.4 C3; 157.4 C5; 134.5 C*ipso* Bn; 132.8 C*p* Bn; 132.2 C*o*′ Bn; 131.0 C*ipso* Ph; 129.3 C*o* Bn; 129.2 C*m*′ Bn; 129.1 C*p* Ph; 128.5 C*m* Ph; 127.7 C*m* Bn; 126.3 C*o* Ph; 48.6 CH_2_Ar; 31.5 CH_2_CH_2_CH_2_CH_2_ *C*H_2_CH_2_CH_3_; 29.1 CH_2_CH_2_CH_2_ *C*H_2_CH_2_CH_2_CH_3_; 28.8 CH_2_CH_2_CH_2_ *C*H_2_CH_2_CH_2_CH_3_; 27.8 CH_2_ *C*H_2_CH_2_CH_2_CH_2_CH_2_CH_3_; 26.0* C*H_2_CH_2_CH_2_CH_2_CH_2_CH_2_CH_3_; 22.5 CH_2_CH_2_CH_2_CH_2_CH_2_ *C*H_2_CH_3_; 14.0 CH_3_. MS (ES^+^)* m/z*: 402 (100%) [M+H]^+^. Anal (C_22_H_25_Cl_2_N_3_) % calculated (% found) C: 65.67 (65.42); H: 6.26 (6.50); N: 10.44 (10.35).** 9b**: ^1^H-NMR (CDCl_3_) *δ*: 7.50–7.43 (m, 5H, Ph); 7.40 (d, 1H, *J* = 1.7 Hz, H*m*′ Bn); 7.20 (dd, 1H, *J* = 8.6 Hz and 1.7 Hz, H*m* Bn); 6.84 (d, 1H, *J* = 8.6 Hz, H*o* Bn); 5.39 (s, 2H, CH_2_Ar); 2.76 (t, 2H, *J* = 7.7 Hz, C*H*_2_CH_2_CH_2_CH_2_CH_2_CH_2_CH_3_); 1.79 (p, 2H, *J* = 7.7 Hz, CH_2_C*H*_2_CH_2_CH_2_CH_2_CH_2_CH_3_); 1.23 (m, 8H, CH_2_CH_2_C*H*_2_C*H*_2_C*H*_2_C*H*_2_CH_3_); 0.85 (bt, 3H, *J* = 6.1 Hz, CH_3_). ^13^C-NMR (CDCl_3_) *δ*: 165.2 C3; 156.2 C5; 134.6 C*ipso* Bn; 133.0 C*p* Bn; 132.9 C*o*′ Bn; 130.5 C*o* Bn; 129.7 C*m*′ Bn; 129.2 C*m* Ph; 129.0 C*p* Ph; 128.6 C*o* Ph; 127.9 C*m* Bn; 127.8 C*ipso* Ph; 50.1 CH_2_Ar; 32.0 CH_2_CH_2_CH_2_CH_2_ *C*H_2_CH_2_CH_3_; 29.6 CH_2_CH_2_ *C*H_2_CH_2_CH_2_CH_2_CH_3_; 29.2 CH_2_CH_2_CH_2_ *C*H_2_CH_2_CH_2_CH_3_; 28.7* C*H_2_ *C*H_2_CH_2_CH_2_CH_2_CH_2_CH_3_; 22.9 CH_2_CH_2_CH_2_CH_2_CH_2_ *C*H_2_CH_3_; 14.3 CH_3_. MS (ES^+^)* m/z*: 402 (100%) [M+H]^+^. Anal (C_22_H_25_Cl_2_N_3_·C_6_H_12_) % calculated (% found) C: 69.12 (69.42); H: 7.67 (7.79); N: 8.64 (8.24).

*1-Benzyl-3-(4-chlorophenyl)-5-heptyl-1H-1,2,4-triazole (* ***10a*** *) and 1-Benzyl-5-(4-chlo-rophenyl)-3-heptyl-1H-1,2,4-triazole (* ***10b***). Compounds** 10a** and** 10b** were prepared from** 4** (80 mg, 0.3 mmol), benzyl bromide (34 *μ*L, 0.3 mmol), and (Bu)_4_NBr (6 mg, 0.02 mmol); reaction time: 20 min. Yield: 94 mg of** 10a** (89%) as a white solid and 9 mg of** 10b** (8%) as a yellow oil.** 10a**: Mp = 47–50°C. ^1^H-NMR (CDCl_3_) *δ*: 8.02 (d, 2H, *J* = 8.8 Hz, H*o* Ar); 7.36 (d, 2H, *J* = 8.8 Hz, H*m* Ar); 7.29 (m, 3H, Bn); 7.19 (m, 2H, Bn); 5.31 (s, 2H, CH_2_Ph); 2.67 (t, 2H, *J* = 7.7 Hz, C*H*_2_CH_2_CH_2_CH_2_CH_2_CH_2_CH_3_); 1.66 (p, 2H, *J* = 7.7 Hz, CH_2_C*H*_2_CH_2_CH_2_CH_2_CH_2_CH_3_); 1.22 (m, 8H, CH_2_CH_2_C*H*_2_C*H*_2_C*H*_2_C*H*_2_CH_3_); 0.85 (bt, 3H, *J* = 6.9 Hz, CH_3_). ^13^C-NMR (CDCl_3_) *δ*: 159.9 C3; 157.1 C5; 135.5 C*ipso* Ar; 134.7 C*ipso* Bn; 129.8 C*p* Ar; 128.9 C*m* Ar; 128.6 C*m* Bn; 128.1 C*p* Bn; 127.6 C*o* Ar; 126.9 C*o* Bn; 52.1 (CH_2_Ph); 31.5 CH_2_CH_2_CH_2_CH_2_ *C*H_2_CH_2_CH_3_; 29.2 CH_2_CH_2_ *C*H_2_CH_2_CH_2_CH_2_CH_3_, 28.8 CH_2_CH_2_CH_2_ *C*H_2_CH_2_CH_2_CH_3_; 27.7 CH_2_ *C*H_2_CH_2_CH_2_CH_2_CH_2_CH_3_; 26.1* C*H_2_CH_2_CH_2_CH_2_CH_2_CH_2_CH_3_; 22.5 CH_2_CH_2_CH_2_CH_2_CH_2_ *C*H_2_CH_3_; 14.0 CH_3_. MS (ES^+^)* m/z*: 368 (100%) [M+H]^+^. Anal (C_22_H_26_ClN_3_) % calculated (% found) C: 71.82 (72.02); H: 7.12 (6.89); N: 11.42 (11.24).** 10b**: ^1^H-NMR (CDCl_3_) *δ*: 7.47 (d, 2H, *J* = 8.5 Hz, H*m* Ar); 7.38 (d, 2H, *J* = 8.5 Hz, H*o* Ar); 7.30 (m, 3H, Bn); 7.00 (m, 2H, Bn); 5.33 (s, 2H, CH_2_Ph); 2.75 (t, 2H, *J* = 7.6 Hz, C*H*_2_CH_2_CH_2_CH_2_CH_2_CH_2_CH_3_); 1.78 (p, 2H, *J* = 7.6 Hz, CH_2_C*H*_2_CH_2_CH_2_CH_2_CH_2_CH_3_); 1.25 (m, 8H, CH_2_CH_2_C*H*_2_C*H*_2_C*H*_2_C*H*_2_CH_3_); 0.85 (bt, 3H, *J* = 6.4 Hz, CH_3_). ^13^C-NMR (CDCl_3_) *δ*: 164.6 C3; 155.0 C5; 136.3 C*ipso* Ar; 136.2 C*ipso* Bn; 130.0 C*m* Ar; 129.4 C*p* Ar; 129.1 C*o* Ar; 129.0 C*m* Bn; 128.0 C*p* Bn; 52.6 (CH_2_Ph); 31.8 CH_2_CH_2_CH_2_CH_2_ *C*H_2_CH_2_CH_3_; 29.4 CH_2_CH_2_ *C*H_2_ *C*H_2_CH_2_CH_2_CH_3_; 29.0 CH_2_ *C*H_2_CH_2_CH_2_CH_2_CH_2_CH_3_; 28.5* C*H_2_CH_2_CH_2_CH_2_CH_2_CH_2_CH_3_; 22.6 CH_2_CH_2_CH_2_CH_2_CH_2_ *C*H_2_CH_3_; 14.0 CH_3_. MS (ES^+^)* m/z*: 368 (100%) [M+H]^+^.

*1-(4-Chlorobenzyl)-3-(4-chlorophenyl)-5-heptyl-1H-1,2,4-triazole (* ***11a*** *) and 1-(4-Chlo-robenzyl)-5-(4-chlorophenyl)-3-heptyl-1H-1,2,4-triazole (* ***11b***). Compounds** 11a** and** 11b** were prepared from** 4** (100 mg, 0.4 mmol), 4-chlorobenzyl chloride (64 mg, 0.4 mmol), and (Bu)_4_NBr (6 mg, 0.02 mmol); reaction time: 6 h. Yield: 132 mg of** 11a** (91%) as a white solid and 2 mg of** 11b** (1%) as a transparent oil.** 11a**: Mp = 58–60°C. ^1^H-NMR (CDCl_3_) *δ*: 8.00 (d, 2H, *J* = 8.8 Hz, H*o* Ar); 7.35 (d, 2H, *J* = 8.8 Hz, H*m* Ar); 7.28 (d, 2H, *J* = 8.4 Hz, H*m* CH_2_Ar); 7.10 (d, 2H, *J* = 8.4 Hz, H*o* CH_2_Ar); 5.26 (s, 2H, CH_2_Ar); 2.66 (t, 2H, *J* = 7.7 Hz, C*H*_2_CH_2_CH_2_CH_2_CH_2_CH_2_CH_3_); 1.66 (p, 2H, *J* = 7.7 Hz, CH_2_C*H*_2_CH_2_CH_2_CH_2_CH_2_CH_3_); 1.23 (m, 8H, CH_2_CH_2_C*H*_2_C*H*_2_C*H*_2_C*H*_2_CH_3_); 0.84 (bt, 3H, *J* = 6.3 Hz, CH_3_). ^13^C-NMR (CDCl_3_) *δ*: 160.1 C3; 157.1 C5; 134.8 C*ipso* Ar; 134.1 C*ipso* CH_2_Ar; 134.0 C*p* CH_2_Ar; 129.6 C*p* Ar; 129.0 C*m* Ar; 128.6 C*m* CH_2_Ar; 128.3 C*o* CH_2_Ar; 127.5 C*o* Ar; 51.3 (CH_2_Ar); 31.5 CH_2_CH_2_CH_2_CH_2_ *C*H_2_CH_2_CH_3_; 29.2 CH_2_CH_2_ *C*H_2_ *C*H_2_CH_2_CH_2_CH_3_; 28.8 CH_2_CH_2_CH_2_ *C*H_2_CH_2_CH_2_CH_3_; 27.7 CH_2_ *C*H_2_CH_2_CH_2_CH_2_CH_2_CH_3_; 26.1* C*H_2_CH_2_CH_2_CH_2_CH_2_CH_2_CH_3_; 22.5 CH_2_CH_2_CH_2_CH_2_CH_2_ *C*H_2_CH_3_; 14.0 CH_3_. MS (ES^+^)* m/z*: 402 (100%) [M+H]^+^. Anal (C_22_H_25_Cl_2_N_3_) % calculated (% found) C: 65.67 (65.72); H: 6.26 (6.12); N: 10.44 (10.40).** 11b**: ^1^H-NMR (CDCl_3_) *δ*: 7.45 (d, 2H, *J* = 9.0 Hz, H*o* Ar); 7.39 (d, 2H, *J* = 9.0 Hz, H*m* Ar); 7.30 (d, 2H, *J* = 8.3 Hz, H*m* CH_2_Ar); 7.05 (d, 2H, *J* = 8.3 Hz, H*o* CH_2_Ar); 5.29 (s, 2H, CH_2_Ar); 2.70 (t, 2H, *J* = 7.8 Hz, C*H*_2_CH_2_CH_2_CH_2_CH_2_CH_2_CH_3_); 1.75 (p, 2H, *J* = 7.8 Hz, CH_2_C*H*_2_CH_2_CH_2_CH_2_CH_2_CH_3_); 1.23 (m, 8H, CH_2_CH_2_C*H*_2_C*H*_2_C*H*_2_C*H*_2_CH_3_); 0.82 (bt, 3H, *J* = 6.2 Hz, CH_3_). ^13^C-NMR (CDCl_3_) *δ*: 164.7 C3; 154.4 C5; 136.4 C*ipso* Ar; 134.4 C*ipso* CH_2_Ar; 134.0 C*p* CH_2_Ar; 129.9 C*m* and C*p* Ar; 129.2 C*o* Ar and C*m* CH_2_Ar; 128.0 C*o* CH_2_Ar; 51.8 CH_2_Ar; 31.8 CH_2_CH_2_CH_2_CH_2_ *C*H_2_CH_2_CH_3_; 29.7 CH_2_CH_2_ *C*H_2_CH_2_CH_2_CH_2_CH_3_; 29.3 CH_2_CH_2_CH_2_ *C*H_2_CH_2_CH_2_CH_3_; 29.0 CH_2_ *C*H_2_CH_2_CH_2_CH_2_CH_2_CH_3_; 28.4* C*H_2_CH_2_CH_2_CH_2_CH_2_CH_2_CH_3_; 22.6 CH_2_CH_2_CH_2_CH_2_CH_2_ *C*H_2_CH_3_; 14.1 CH_3_. MS (ES^+^)* m/z*: 402 (100%) [M+H]^+^. HPLC: Acetonitrile/H_2_O 95 : 5, *t*_*R*_ = 25.5 min (81% purity).

*3-(4-Chlorophenyl)-1-(2,4-dichlorobenzyl)-5-heptyl-1H-1,2,4-triazole (* ***12a*** *) and 5-(4-Chlorophenyl)-1-(2,4-dichlorobenzyl)-3-heptyl-1H-1,2,4-triazole (* ***12b***). Compounds** 12a** and** 12b** were prepared from** 4** (90 mg, 0.3 mmol), 2,4-dichlorobenzyl chloride (45 *μ*L, 0.3 mmol), and (Bu)_4_NBr (6 mg, 0.02 mmol); reaction time: 6 h. Yield: 122 mg of** 12a** (86%) as a white solid and 8 mg of** 12b** (6%) as a transparent oil.** 12a**: Mp = 97–99°C. ^1^H-NMR (CDCl_3_) *δ*: 8.00 (d, 2H, *J* = 8.6 Hz, H*o* Ar); 7.41 (d, 1H, *J* = 2.0 Hz, H*m*′ CH_2_Ar); 7.37 (d, 2H, *J* = 8.6 Hz, H*m* Ar); 7.18 (dd, 1H, *J* = 8.4 Hz and 2.0 Hz, H*m* CH_2_Ar); 6.84 (d, 1H, *J* = 8.4 Hz, H*o* CH_2_Ar); 5.37 (s, 2H, CH_2_Ar); 2.70 (t, 2H, *J* = 7.6 Hz, C*H*_2_CH_2_CH_2_CH_2_CH_2_CH_2_CH_3_); 1.69 (p, 2H, *J* = 7.6 Hz, CH_2_C*H*_2_CH_2_CH_2_CH_2_CH_2_CH_3_); 1.22 (m, 8H, CH_2_CH_2_C*H*_2_C*H*_2_C*H*_2_C*H*_2_CH_3_); 0.85 (bt, 3H, *J* = 7.1 Hz, CH_3_). ^13^C-NMR (CDCl_3_) *δ*: 160.5 C3; 157.6 C5; 135.0 C*ipso* Ar; 134.6 C*ipso* CH_2_Ar; 132.9 C*p* CH_2_Ar; 132.0 C*o*′ CH_2_Ar; 129.5 C*p* Ar; 129.4 C*o* CH_2_Ar; 129.3 C*m*′ CH_2_Ar; 128.7 C*m* Ar; 127.7 C*m* CH_2_Ar; 127.6 C*o* Ar; 48.7 CH_2_Ar; 31.5 CH_2_CH_2_CH_2_CH_2_ *C*H_2_CH_2_CH_3_; 29.1 CH_2_CH_2_ *C*H_2_ *C*H_2_CH_2_CH_2_CH_3_; 28.8 CH_2_CH_2_CH_2_ *C*H_2_CH_2_CH_2_CH_3_; 27.7 CH_2_ *C*H_2_CH_2_CH_2_CH_2_CH_2_CH_3_; 26.0* C*H_2_CH_2_CH_2_CH_2_CH_2_CH_2_CH_3_; 22.5 CH_2_CH_2_CH_2_CH_2_CH_2_ *C*H_2_CH_3_; 14.0 CH_3_. MS (ES^+^)* m/z*: 436 (100%) [M+H]^+^. Anal (C_22_H_24_Cl_3_N_3_) % calculated (% found) C: 60.49 (60.42); H: 5.54 (5.74); N: 9.62 (9.42).** 12b**: ^1^H-NMR (CDCl_3_) *δ*: 7.41 (bs, 5H, H*o* Ar, H*m* Ar and H*m*′ CH_2_Ar); 7.21 (dd, 1H, *J* = 8.3 Hz and 2.0 Hz, H*m* CH_2_Ar); 6.84 (d, 1H, *J* = 8.3 Hz, H*o* CH_2_Ar); 5.37 (s, 2H, CH_2_Ar); 2.75 (t, 2H, *J* = 7.7 Hz, C*H*_2_CH_2_CH_2_CH_2_CH_2_CH_2_CH_3_); 1.77 (p, 2H, *J* = 7.7 Hz, CH_2_C*H*_2_CH_2_CH_2_CH_2_CH_2_CH_3_); 1,23 (m, 8H, CH_2_CH_2_C*H*_2_C*H*_2_C*H*_2_C*H*_2_CH_3_); 0.85 (bt, 3H, *J* = 6.1 Hz, CH_3_). ^13^C-NMR (CDCl_3_) *δ*: 165.1 C3; 154.9 C5; 136.6 C*ipso* Ar; 134.7 C*ipso* CH_2_Ar; 132.8 C*p* CH_2_Ar; 132.5 C*o*′ CH_2_Ar; 129.7 C*m* and C*p* Ar; 129.6 C*o* CH_2_Ar; 129.3 C*o* Ar; 129.0 C*m*′ CH_2_Ar; 127.8 C*m* CH_2_Ar; 49.9 CH_2_Ar; 31.8 CH_2_CH_2_CH_2_CH_2_ *C*H_2_CH_2_CH_3_; 29.7 CH_2_CH_2_ *C*H_2_ *C*H_2_CH_2_CH_2_CH_3_; 29.3 CH_2_CH_2_CH_2_ *C*H_2_CH_2_CH_2_CH_3_; 29.0 CH_2_ *C*H_2_CH_2_CH_2_CH_2_CH_2_CH_3_; 28.4* C*H_2_CH_2_CH_2_CH_2_CH_2_CH_2_CH_3_; 22.6 CH_2_CH_2_CH_2_CH_2_CH_2_ *C*H_2_CH_3_; 14.0 CH_3_. MS (ES^+^)* m/z*: 436 (99%) [M+H]^+^. HPLC: Acetonitrile/H_2_O 90 : 10, *t*_*R*_ = 66.2 min (99% purity).

*4-(1-Benzyl-5-heptyl-1H-1,2,4-triazol-3-yl)pyridine (* ***13a***). Compound** 13a** was prepared from** 5** (150 mg, 0.6 mmol), benzyl chloride (73 *μ*L, 0.6 mmol), and (Bu)_4_NBr (6 mg, 0.02 mmol); reaction time: 2.5 h. Yield: 166 mg of** 13a** (81%) as an orange oil. ^1^H-NMR (CDCl_3_) *δ*: 8.63 (d, 2H, *J* = 6.0 Hz, H*m* pyr); 7.93 (d, 2H, *J* = 6.0 Hz, H*o* pyr); 7.31 (m, 3H, Ph); 7.16 (m, 2H, Ph); 5.33 (s, 2H, CH_2_Ar); 2.68 (t, 2H, *J* = 7.8 Hz, C*H*_2_CH_2_CH_2_CH_2_CH_2_CH_2_CH_3_); 1.65 (p, 2H, *J* = 7.0 Hz, CH_2_C*H*_2_CH_2_CH_2_CH_2_CH_2_CH_3_); 1.21 (m, 8H, CH_2_CH_2_C*H*_2_C*H*_2_C*H*_2_C*H*_2_CH_3_); 0.83 (bt, 3H, *J* = 7.1 Hz, CH_3_). ^13^C-NMR (CDCl_3_) *δ*: 158.7 C3; 157.5 C5; 150.1 C*m* pyr; 138.5 C*ipso* pyr; 135.2 C*ipso* Ph; 128.9 C*m* Ph; 128.2 C*p* Ph; 127.0 C*o* Ph; 120.4 C*o* pyr; 52.3 CH_2_Ph; 31.5 CH_2_CH_2_CH_2_CH_2_ *C*H_2_CH_2_CH_3_; 29.1 CH_2_CH_2_ *C*H_2_ *C*H_2_CH_2_CH_2_CH_3_; 28.8 CH_2_CH_2_CH_2_ *C*H_2_CH_2_CH_2_CH_3_; 27.6 CH_2_ *C*H_2_CH_2_CH_2_CH_2_CH_2_CH_3_; 26.1* C*H_2_CH_2_CH_2_CH_2_CH_2_CH_2_CH_3_; 22.5 CH_2_CH_2_CH_2_CH_2_CH_2_ *C*H_2_CH_3_; 14.0 CH_3_. MS (ES^+^)* m/z*: 335 (100%) [M+H]^+^. Anal (C_21_H_26_N_4_) % calculated (% found) C: 75.41 (75.71); H: 7.84 (7.79); N: 16.75 (16.54).

*4-[1-(4-Chlorobenzyl)-5-heptyl-1H-1,2,4-triazol-3-yl]pyridine (* ***14a*** *) and 4-[1-(4-Chlo-robenzyl)-3-heptyl-1H-1,2,4-triazol-5-yl]pyridine (* ***14b***). Compounds** 14a** and** 14b** were prepared from** 5** (180 mg, 0.7 mmol), 4-chlorobenzyl chloride (119 mg, 0.7 mmol), and (Bu)_4_NBr (12 mg, 0.04 mmol); reaction time: 6 h. Yield: 150 mg of** 14a** (51%) as a brown solid and 10 mg of** 14b** (4%) as a brown oil.** 14a**: Mp = 77–80°C. ^1^H-NMR (CDCl_3_) *δ*: 8.61 (d, 2H, *J* = 6.0 Hz, H*m* pyr); 7.90 (d, 2H, *J* = 6.0 Hz, H*o* pyr); 7.27 (d, 2H, *J* = 8.6 Hz, H*m* Ar); 7.09 (d, 2H, *J* = 8.6 Hz, H*o* Ar); 5.27 (s, 2H, CH_2_Ar); 2.66 (t, 2H, *J* = 7.8 Hz, C*H*_2_CH_2_CH_2_CH_2_CH_2_CH_2_CH_3_); 1.65 (p, 2H, *J* = 7.8 Hz, CH_2_C*H*_2_CH_2_CH_2_CH_2_CH_2_CH_3_); 1.20 (m, 8H, CH_2_CH_2_C*H*_2_C*H*_2_C*H*_2_C*H*_2_CH_3_); 0.82 (bt, 3H, *J* = 6.6 Hz, CH_3_). ^13^C-NMR (CDCl_3_) *δ*: 158.9 C3; 157.5 C5; 150.2 C*m* pyr; 138.4 C*ipso* pyr; 134.2 C*ipso* Ar; 133.7 C*p* Ar; 129.1 C*m* Ar; 128.4 C*o* Ar; 120.4 C*o* pyr; 51.8 CH_2_Ar; 31.5 CH_2_CH_2_CH_2_CH_2_ *C*H_2_CH_2_CH_3_; 29.1 CH_2_CH_2_ *C*H_2_ *C*H_2_CH_2_CH_2_CH_3_; 28.8 CH_2_CH_2_CH_2_ *C*H_2_CH_2_CH_2_CH_3_; 26.0* C*H_2_CH_2_CH_2_CH_2_CH_2_CH_2_CH_3_; 22.5 CH_2_CH_2_CH_2_CH_2_CH_2_ *C*H_2_CH_3_; 14.0 CH_3_. MS (ES^+^)* m/z*: 369 (100%) [M+H]^+^. Anal (C_21_H_25_ClN_4_) % calculated (% found) C: 68.37 (68.55); H: 6.83 (6.90); N: 15.19 (15.21).** 14b**: ^1^H-NMR (CDCl_3_) *δ*: 8.65 (d, 2H, *J* = 6.2 Hz, H*m* pyr); 7.44 (d, 2H, *J* = 6.2 Hz, H*o* pyr); 7.31 (d, 2H, *J* = 8.5 Hz, H*m* Ar); 7.06 (d, 2H, *J* = 8.5 Hz, H*o* pyr); 5.35 (s, 2H, CH_2_Ar); 2.76 (t, 2H, *J* = 7.6 Hz, C*H*_2_CH_2_CH_2_CH_2_CH_2_CH_2_CH_3_); 1.74 (p, 2H, *J* = 7.6 Hz, CH_2_C*H*_2_CH_2_CH_2_CH_2_CH_2_CH_3_); 1.27 (m, 8H, CH_2_CH_2_C*H*_2_C*H*_2_C*H*_2_C*H*_2_CH_3_); 0.85 (m, 3H, CH_3_). ^13^C-NMR (CDCl_3_) *δ*: 165.1 C3; 152.8 C5; 150.5 C*m* pyr; 135.4 C*ipso* pyr; 134.2 C*ipso* Ar; 134.0 C*p* Ar; 129.3 C*m* Ar; 128.0 C*o* Ar; 122.5 C*o* pyr; 52.1 CH_2_Ar; 31.7 CH_2_CH_2_CH_2_CH_2_ *C*H_2_CH_2_CH_3_; 29.3 CH_2_CH_2_ *C*H_2_ *C*H_2_CH_2_CH_2_CH_3_; 29.2 CH_2_CH_2_CH_2_ *C*H_2_CH_2_CH_2_CH_3_; 28.4 and 29.0* C*H_2_ *C*H_2_CH_2_CH_2_CH_2_CH_2_CH_3_; 22.6 CH_2_CH_2_CH_2_CH_2_CH_2_ *C*H_2_CH_3_; 14.1 CH_3_. MS (ES^+^)* m/z*: 369 (100%) [M+H]^+^.

*4-[1-(2,4-Dichlorobenzyl)-5-heptyl-1H-1,2,4-triazol-3-yl]pyridine (* ***15a*** *) and 4-[1-(2,4-Dichlorobenzyl)-3-heptyl-1H-1,2,4-triazol-5-yl]pyridine (* ***15b***). Compounds** 15a** and** 15b** were prepared from** 5** (200 mg, 0.8 mmol), 2,4-dichlorobenzyl chloride (114 *μ*L, 0.8 mmol), and (Bu)_4_NBr (12 mg, 0.04 mmol.); reaction time: 2 h. Yield: 118 mg of** 15a** (36%) as a white solid and 7 mg of** 15b** (2%) as a yellow oil.** 15a**: Mp = 104-105°C. ^1^H-NMR (CDCl_3_) *δ*: 8.65 (d, 2H, *J* = 6.1 Hz, H*m* pyr); 7.93 (d, 2H, *J* = 6.1 Hz, H*o* pyr); 7.42 (d, 1H, *J* = 2.2 Hz, H*m*′ Ar); 7.19 (dd, 1H, *J* = 8.4 Hz and 2.2 Hz, H*m* Ar); 6.58 (d, 1H, *J* = 8.4 Hz, H*o* Ar); 5.39 (s, 2H, CH_2_Ar); 2.72 (t, 2H, *J* = 7.7 Hz, C*H*_2_CH_2_CH_2_CH_2_CH_2_CH_2_CH_3_); 1.70 (p, 2H, *J* = 7.7 Hz, CH_2_C*H*_2_CH_2_CH_2_CH_2_CH_2_CH_3_); 1.23 (m, 8H, CH_2_CH_2_C*H*_2_C*H*_2_C*H*_2_C*H*_2_CH_3_); 0.84 (bt, 3H, *J* = 6.5 Hz, CH_3_). ^13^C-NMR (CDCl_3_) *δ*: 159.3 C3; 158.1 C5; 150.3 C*m* pyr; 138.3 C*ipso* pyr; 134.8 C*ipso* Ar; 133.0 C*p* Ar; 131.7 C*o*′ Ar; 129.5 C*o* and C*m*′ Ar; 127.8 C*m* Ar; 120.4 C*o* pyr; 48.9 (CH_2_Ar); 31.6 CH_2_CH_2_CH_2_CH_2_ *C*H_2_CH_2_CH_3_; 29.1 CH_2_CH_2_ *C*H_2_ *C*H_2_CH_2_CH_2_CH_3_; 28.8 CH_2_CH_2_CH_2_ *C*H_2_CH_2_CH_2_CH_3_; 27.7 CH_2_ *C*H_2_CH_2_CH_2_CH_2_CH_2_CH_3_; 26.0* C*H_2_CH_2_CH_2_CH_2_CH_2_CH_2_CH_3_; 22.5 CH_2_CH_2_CH_2_CH_2_CH_2_ *C*H_2_CH_3_; 14.0 CH_3_. MS (ES^+^)* m/z*: 403 (100%) [M+H]^+^. Anal (C_21_H_24_Cl_2_N_4_) % calculated (% found) C: 62.53 (62.28); H: 6.00 (6.30); N: 13.89 (14.04).** 15b**: ^1^H-NMR (CDCl_3_) *δ*: 8.70 (d, 2H, *J* = 6.1 Hz, H*m* pyr); 7.43 (d, 1H, *J* = 2.2 Hz, H*m*′ Ar); 7.41 (d, 2H, *J* = 6.1 Hz, H*o* pyr); 7.19 (dd, 1H, *J* = 8.1 Hz and 2.2 Hz, H*m* Ar); 6.85 (d, 1H, *J* = 8.1 Hz, H*o* Ar); 5.44 (s, 2H, CH_2_Ar); 2.77 (t, 2H, *J* = 7.6 Hz, C*H*_2_CH_2_CH_2_CH_2_CH_2_CH_2_CH_3_); 1.73 (p, 2H, *J* = 7.6 Hz, CH_2_C*H*_2_CH_2_CH_2_CH_2_CH_2_CH_3_); 1.25 (m, 8H, CH_2_CH_2_C*H*_2_C*H*_2_C*H*_2_C*H*_2_CH_3_); 0.85 (ta, 3H, *J* = 6.7 Hz, CH_3_). ^13^C-NMR (CDCl_3_) *δ*: 165.5 C3; 152.8 C5; 150.6 C*m* pyr; 138.3 C*ipso* pyr; 135.1 C*ipso* Ar; 132.8 C*p* Ar; 131.9 (C*o*′ Ar); 129.7 C*o* Ar; 128.8 C*m*′ Ar; 127.8 C*m* Ar; 122.3 C*o* pyr; 50.1 CH_2_Ar; 31.6 CH_2_CH_2_CH_2_CH_2_ *C*H_2_CH_2_CH_3_; 29.3 CH_2_CH_2_ *C*H_2_ *C*H_2_CH_2_CH_2_CH_3_; 29.0 CH_2_CH_2_CH_2_ *C*H_2_CH_2_CH_2_CH_3_; 28.3* C*H_2_ *C*H_2_CH_2_CH_2_CH_2_CH_2_CH_3_; 22.6 CH_2_CH_2_CH_2_CH_2_CH_2_ *C*H_2_CH_3_; 14.1 CH_3_. MS (ES^+^)* m/z*: 403 (100%) [M+H]^+^. HPLC: Acetonitrile/H_2_O 90 : 10, *t*_*R*_ = 30.3 min (90% purity).

#### 2.1.5. General Procedure for the Synthesis of** 16**–**18**

To a solution of the corresponding triazole (1 equiv) in dry CH_2_Cl_2_ (4–10 mL), excess of MeI was added. The reaction mixture was stirred at room temperature for the time indicated. Afterwards, solvent was removed* in vacuo* and the residue was purified by chromatography or recrystallization from Et_2_O/CH_2_Cl_2_.

*4-(1-Benzyl-5-heptyl-1H-1,2,4-triazol-3-yl)-1-methylpyridinium Iodide (* ***16***). Compound** 16** was prepared from** 13a** (15 mg, 0.05 mmol) and MeI (4 *μ*L, 0.07 mmol); reaction time: 16 h. Purification: flash chromatography [CH_2_Cl_2_/MeOH (99 : 1) → CH_2_Cl_2_/MeOH (9 : 1)]. Yield: 14 mg of** 16** (66%) as a yellow gummy solid. ^1^H-NMR (CDCl_3_) *δ*: 9.18 (d, 2H, *J* = 6.7 Hz, H*m* pyr); 8.54 (d, 2H, *J* = 6.7 Hz, H*o* pyr); 7.35 (m, 3H, Ph); 7.19 (m, 2H, Ph); 5.36 (s, 2H, CH_2_Ar); 4.68 (s, 3H, NMe); 2.71 (t, 2H, *J* = 7.6 Hz, C*H*_2_CH_2_CH_2_CH_2_CH_2_CH_2_CH_3_); 1.67 (p, 2H, *J* = 7.6 Hz, CH_2_C*H*_2_CH_2_CH_2_CH_2_CH_2_CH_3_); 1.23 (m, 8H, CH_2_CH_2_C*H*_2_C*H*_2_C*H*_2_C*H*_2_CH_3_); 0.85 (bt, 3H, *J* = 7.0 Hz, CH_3_). ^13^C-NMR (CDCl_3_) *δ*: 159.0 C3; 155.3 C5; 146.7 C*ipso* pyr; 145.6 C*m* pyr; 134.3 C*ipso* Ph; 129.1 C*m* Ph; 128.6 C*p* Ph; 127.3 C*o* Ph; 123.7 C*o* pyr; 53.0 CH_2_Ar; 49.1 NMe; 31.5 CH_2_CH_2_CH_2_CH_2_ *C*H_2_CH_2_CH_3_; 29.0 CH_2_CH_2_ *C*H_2_ *C*H_2_CH_2_CH_2_CH_3_; 28.8 CH_2_CH_2_CH_2_ *C*H_2_CH_2_CH_2_CH_3_; 27.2 CH_2_ *C*H_2_CH_2_CH_2_CH_2_CH_2_CH_3_; 26.1* C*H_2_CH_2_CH_2_CH_2_CH_2_CH_2_CH_3_; 22.5 CH_2_CH_2_CH_2_CH_2_CH_2_ *C*H_2_CH_3_; 14.0 CH_3_. MS (ES^+^)* m/z*: 349 (100%), [M]^+^. Anal (C_22_H_29_IN_4_) % calculated (% found) C: 55.47 (55.42); H: 6.14 (6.30); N: 11.76 (11.57).

*4-[1-(4-Chlorobenzyl)-5-heptyl-1H-1,2,4-triazol-3-yl]-1-methylpyridinium Iodide (* ***17***). Compound** 17** was prepared from** 14a** (70 mg, 0.2 mmol) and MeI (140 *μ*L 2.3 mmol); reaction time: 8 days. Purification: flash chromatography [CH_2_Cl_2_/MeOH (95 : 5)]. Yield: 87 mg of** 17** (90%) as a yellow gummy solid. ^1^H-NMR (CDCl_3_) *δ*: 9.21 (d, 2H, *J* = 6.8 Hz, H*m* pyr); 8.54 (d, 2H, *J* = 6.8 Hz, H*o* pyr); 7.33 (d, 2H, *J* = 8.5 Hz, H*m* Ar); 7.15 (d, 2H, *J* = 8.5 Hz, H*o* Ar); 5.33 (s, 2H, CH_2_Ar); 4.68 (s, 3H, NMe); 2.71 (t, 2H, *J* = 7.5 Hz, C*H*_2_CH_2_CH_2_CH_2_CH_2_CH_2_CH_3_); 1.69 (p, 2H, *J* = 7.5 Hz, CH_2_C*H*_2_CH_2_CH_2_CH_2_CH_2_CH_3_); 1.23 (m, 8H, CH_2_CH_2_C*H*_2_C*H*_2_C*H*_2_C*H*_2_CH_3_); 0.85 (bt, 3H, *J* = 6.5 Hz, CH_3_). ^13^C-NMR (CDCl_3_) *δ*: 158.9 C3; 155.4 C5; 146.3 C*ipso* pyr; 145.7 C*m* pyr; 134.4 C*ipso* Ar; 132.7 C*p* Ar; 129.1 C*m* Ar; 128.7 C*o* Ar; 123.5 C*o* pyr; 52.1 CH_2_Ar; 48.9 NMe; 31.4 CH_2_CH_2_CH_2_CH_2_ *C*H_2_CH_2_CH_3_; 28.9 CH_2_CH_2_ *C*H_2_ *C*H_2_CH_2_CH_2_CH_3_; 28.6 CH_2_CH_2_CH_2_ *C*H_2_CH_2_CH_2_CH_3_; 27.0 CH_2_ *C*H_2_CH_2_CH_2_CH_2_CH_2_CH_3_; 25.9* C*H_2_CH_2_CH_2_CH_2_CH_2_CH_2_CH_3_; 22.4 CH_2_CH_2_CH_2_CH_2_CH_2_ *C*H_2_CH_3_; 13.9 CH_3_. MS (ES^+^)* m/z*: 383 (100%) [M]^+^. Anal (C_22_H_28_ClIN_4_) % calculated (% found) C: 51.73 (51.58); H: 5.52 (5.41); N: 10.97 (10.72).

*4-[1-(2,4-Dichlorobenzyl)-5-heptyl-1H-1,2,4-triazol-3-yl]-1-methylpyridinium Iodide (* ***18***). Compound** 18** was prepared from** 15a** (50 mg, 0.1 mmol) and MeI (277 *μ*L 4.45 mmol); reaction time: 8 days. Purification: recrystallization from Et_2_O/CH_2_Cl_2_. Yield: 43 mg of** 18** (64%) as a yellow solid. Mp = 136–138°C. ^1^H-NMR (CDCl_3_) *δ*: 9.22 (d, 2H, *J* = 6.7 Hz, H*m* pyr); 8.53 (d, 2H, *J* = 6.7 Hz, H*o* pyr); 7.44 (d, 1H, *J* = 1.9 Hz, H*m*′ Ar); 7.25 (dd, 1H, *J* = 8.4 Hz and 1.9 Hz, H*m* Ar); 7.00 (d, 1H, *J* = 8.4 Hz, H*o* Ar); 5.42 (s, 2H, CH_2_Ar); 4.69 (s, 3H, NMe); 2.76 (t, 2H, *J* = 7.6 Hz, C*H*_2_CH_2_CH_2_CH_2_CH_2_CH_2_CH_3_); 1.72 (p, 2H, *J* = 7.6 Hz, CH_2_C*H*_2_CH_2_CH_2_CH_2_CH_2_CH_3_); 1.26 (m, 8H, CH_2_CH_2_C*H*_2_C*H*_2_C*H*_2_C*H*_2_CH_3_); 0.86 (bt, 3H, *J* = 7.3 Hz, CH_3_). ^13^C-NMR (CDCl_3_) *δ*: 159.4 C3; 155.8 C5; 146.5 C*ipso* pyr; 145.7 C*m* pyr; 135.4 C*ipso* Ar; 133.4 C*p* Ar; 130.6 C*o*′ Ar; 130.3 C*o* Ar; 129.7 C*m*′ Ar; 128,0 (C*m Bn*Ar); 123,7 (C*o C*Ar); 49,5 (CH_2_Ar); 49,2 (NMe); 31.5 CH_2_CH_2_CH_2_CH_2_ *C*H_2_CH_2_CH_3_; 29.0 CH_2_CH_2_ *C*H_2_ *C*H_2_CH_2_CH_2_CH_3_; 28.8 CH_2_CH_2_CH_2_ *C*H_2_CH_2_CH_2_CH_3_; 27.3 CH_2_ *C*H_2_CH_2_CH_2_CH_2_CH_2_CH_3_; 25.9* C*H_2_CH_2_CH_2_CH_2_CH_2_CH_2_CH_3_; 22.5 CH_2_CH_2_CH_2_CH_2_CH_2_ *C*H_2_CH_3_; 14.0 CH_3_. MS (ES^+^)* m/z*: 417 (100%) [M]^+^. Anal (C_22_H_27_Cl_2_IN_4_) % calculated (% found) C: 48.46 (48.71); H: 4.99 (5.20); N: 10.27 (10.06).

### 2.2. Pharmacology

*Radioligand Binding Assays*. CB_1_R binding assays in rat cerebellar membranes were performed using [^3^H]-SR141716A and [^3^H]-WIN552122 (NEN-Dupont, Boston, MA, 40–60 Ci/mmol) as radioligands, using the previously described methods [14]. *K*_*i*_ were calculated from the equation of Yung-Chi and Prusoff [15], using fixed *K*_*d*_ values for either [^3^H]-WIN552122 (8 nM) or [^3^H]-SR141716A (4 nM) obtained from independent experimental assays.

## 3. Results and Discussion

### 3.1. Chemistry

**Scheme 2:**
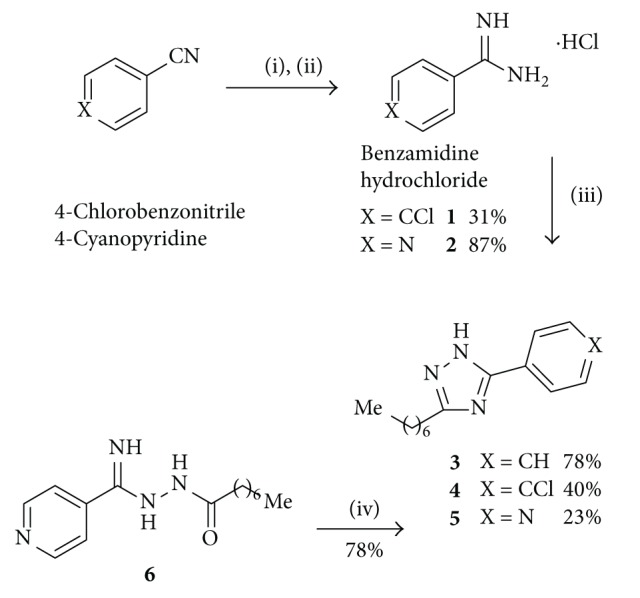
Reagents: (i) NaOMe, dry EtOH, N_2 _atm; (ii) NH_4_Cl, N_2 _atm; (iii) octanoic hydrazide, NaOMe, dry EtOH, Δ; and (iv) NaOMe, dry EtOH, Δ.

5-Aryl-3-heptyl-1*H*-1,2,4-triazoles were first synthesized, and then they were alkylated with different benzyl halide reagents. Preparation of disubstituted triazoles** 3**–**5** is depicted in Scheme 2. In the first step, 4-chlorobenzonitrile and 4-cyanopyridine reacted successively with sodium methoxide and ammonium chloride under inert conditions to afford amidinium hydrochlorides** 1** and** 2**, respectively. Triazoles** 3**–**5** were obtained from** 1**,** 2** and the commercially available benzamidine hydrochloride in moderate yields by refluxing them with octanoic hydrazide under basic conditions. Cyclization of 4-amidinopyridinium hydrochloride (**2**) was incomplete and the addition intermediate** 6** was allowed to be isolated. Acylamidrazone** 6** was then cyclized to** 5** under the same basic conditions (Scheme 2).

**Scheme 3:**
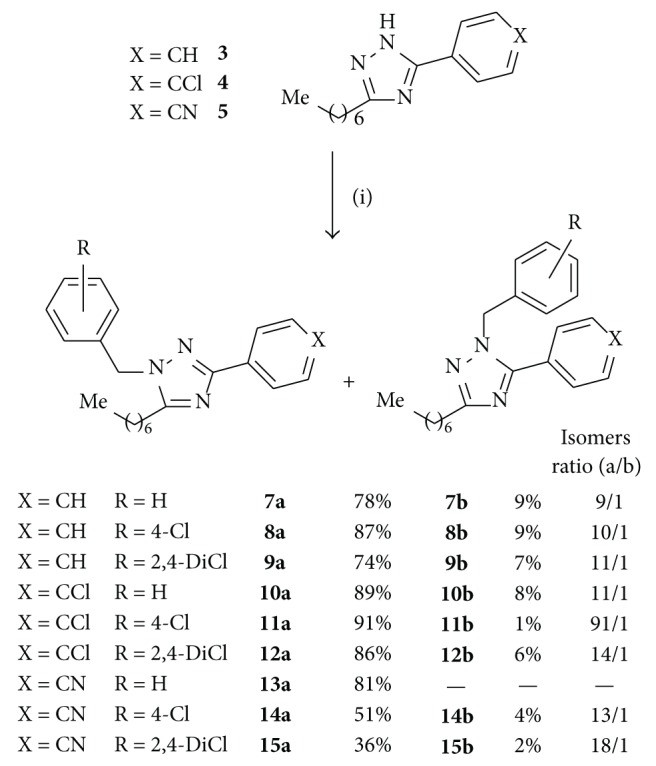
Reagents: (i) benzyl bromide for** 7a**-**7b**,** 10a**-**10b**, and** 13a**; 4-chlorobenzyl chloride for** 8a**-**8b**,** 11a**-**11b**, and** 14a**-**14b**; and 2,4-dichlorobenzyl chloride for** 9a**-**9b**,** 12a**-**12b**, and** 15a**-**15b**; 40% NaOH aq, Bu_4_NBr, and toluene, 80°C.

The second step took place with the alkylation of triazoles** 3**–**5** under phase transfer catalysis conditions, using an aqueous sodium hydroxide solution as base and toluene as organic solvent [16]. These conditions were chosen after unsuccessful attempts of alkylation in an organic solvent (tetrahydrofuran) with mild (sodium bicarbonate) or strong (sodium hydride) bases. As depicted in Scheme 3, reaction of** 3**–**5** with different benzyl halides in the presence of tetrabutylammonium bromide yielded two products by alkylation on N2 (**7a**–**15a**) or N1 (**7b**–**15b**) of the triazole. Alkylation on N4 of the triazole was not detected, since its formation is hindered by steric reasons. Both alkylated isomers were easily isolated by chromatography, being the N2-benzyl derivatives obtained in greater proportion (≈10 : 1). The only N1 isomer that could not be isolated and characterized was** 13b**; however it was detected by HPLC during the synthesis of** 13a**. Higher ratio of N2 isomers was obtained by alkylation of** 5** with 4-chlorobenzyl and 2,4-dichlorobenzyl chlorides that led to a mixture of N2/N1 isomers in proportion of 13 : 1 and 18 : 1, respectively. These results support the fact that alkylation of 1,2,4-triazoles with benzyl halides is governed by steric reasons.

**Scheme 4:**
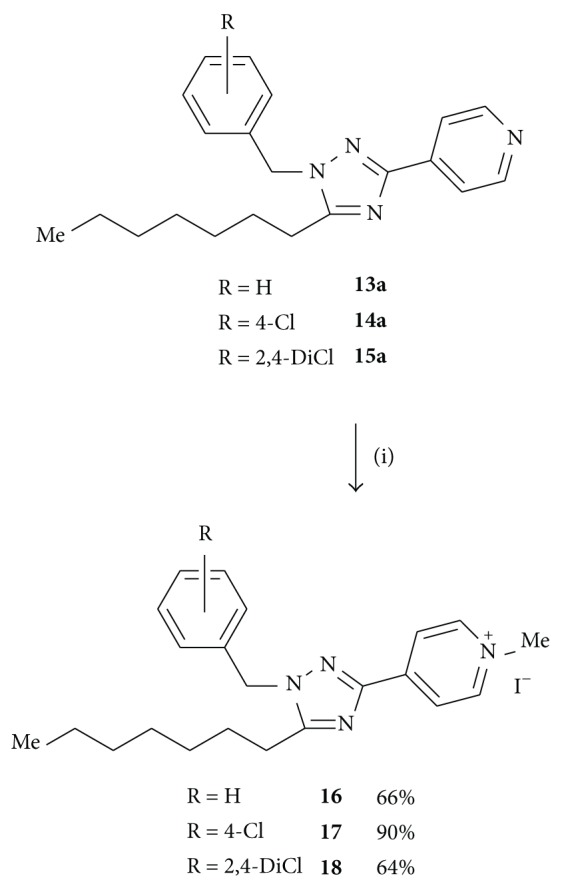
Reagents: (i) MeI (excess), CH_2_Cl_2_, rt.

Since compounds** 7**–**15** are very lipophilic, pyridinium salts (**16**–**18**; Scheme 4) of some of the triazolylpyridines previously obtained were synthesized in order to test if they possessed improved aqueous solubility compared to the parent compounds. Increasing the aqueous solubility was important to perform the radioligand binding assays of the series of benzyl triazoles. Therefore, compounds** 13a**–**15a** readily reacted with an excess of methyl iodide (1.5 equiv for** 13a**, 11 equiv for** 14a**, and 44 equiv for** 15a**). Achievement of the triazolyl-1-methyl pyridinium salts needed long reaction times (16 h for** 16** and 8 days for** 17** and** 18**), but the products were obtained in good yields.

Qualitative solubility tests of compounds** 16**–**18** did not show any improvement in their solubility in water; therefore they were not assessed by pharmacological assays.

### 3.2. Radioligand Binding Assays

Competitive radioligand binding assays have been used to evaluate the affinity of selected synthetized triazoles to CB_1_R in rat cerebellar membranes. They have been performed with [^3^H]-SR141716A and [^3^H]-WIN552122 as labelled ligands. The results of these preliminary assays are reported in Table 1.

Compound** 12a** showed high CB_1_R affinity versus [^3^H]-SR141617 (*K*_*i*_ = 13.9 nM) and moderate affinity versus [^3^H]-WIN552122 (*K*_*i*_ = 323 nM). These binding data indicate that** 12a** displaced better SR141617, an inverse agonist of CB_1_R, than WIN552122, an agonist of CB_1_R. Since both SR141716 and WIN552122 have been reported in the literature to bind to CB_1_R in the same binding pocket [17], the results obtained here suggest that** 12a** binds to the inactive state of CB_1_R, as the inverse agonists do (e.g., SR141716), and not to the active state of the receptor, as the agonists do (e.g., WIN552122) [18].

The other tested compounds** 7a**,** 8a**,** 10a**, and** 11a** showed moderate CB_1_R affinity with affinity constant values in the low micromolar range.

In what refers to the binding to CB_2_R, none of the compounds showed significant affinity using [^3^H]-CP55940 as radioligand in membranes purified from cells transfected with human CB_2_R (data not shown).

## 4. Conclusions

In our ongoing program searching for novel cannabinoid ligands, we reported a CB_1_R antagonist [5-(4-chlorophenyl)-1-(2,4-dichlorophenyl)-3-hexyl-1*H*-1,2,4-triazole, LH21], which showed an interesting* in vitro* and* in vivo* pharmacological profile and was able to reduce food intake and body weight in obese animals with major peripheral components. In the present study, we have explored structural modifications on this 1,2,4-triazole scaffold. A series of new 3(5)-alkyl-5(3)-aryl-1-benzyl-1*H*-1,2,4-triazoles were synthesized and competitive binding assays of selected compounds were carried out. One of these triazoles (**12a**) showed high affinity for CB_1_R.

## Figures and Tables

**Table 1:** Affinity of compounds **7a**-**8a **and** 10a–12a** and the reference cannabinoids SR141716 and LH21 for CB_1_R determined using rat cerebellar membranes and [^3^H]-SR141716 or [^3^H]-WIN552122 as radioligand. *K*_*i*_ values were obtained from three independent experiments carried out in triplicate and are expressed as mean ± standard error.

Compound	*K*_*i*_ (nM) CB_1_R versus [^3^H]-SR141617	*K*_*i*_ (nM) CB_1_R versus [^3^H]-WIN552122
SR141716	*K*_*d*_ = 0.59	4
LH21	855.6 ± 296	748 ± 193
**7a**	436 ± 120	477 ± 94
**8a**	589 ± 136	561 ± 125
**10a**	389.5 ± 180	2437 ± 888
**11a**	562 ± 183	720 ± 165
**12a**	13.9 ± 2.4	323 ± 60.5
